# Serial personal digital assistant data capture of health-related quality of life: A randomized controlled trial in a prostate cancer clinic

**DOI:** 10.1186/1477-7525-5-38

**Published:** 2007-07-06

**Authors:** Andrew G Matthew, Kristen L Currie, Jane Irvine, Paul Ritvo, Daniel Santa Mina, Leah Jamnicky, Robert Nam, John Trachtenberg

**Affiliations:** 1 Department of Surgical Oncology, University Health Network, Toronto, Ontario, Canada; 2 Department of Surgery, University of Toronto, Toronto, Ontario, Canada; 3 Department of Psychology, York University, 4700 Keele St, Toronto, Ontario M3J 1P3, Canada; 4 School of Kinesiology & Health Science, York University, 4700 Keele St, Toronto, Ontario M3J 1P3, Canada; 5 Division of Preventive Oncology, Cancer Care Ontario, 620 University Avenue, Toronto, Ontario, M5G 2M9, Canada; 6 Divisions of Urology and Surgical Oncology, Sunnybrook Health Sciences Centre, 2075 Bayview Avenue, Toronto, Ontario, M4N 3M5, Canada

## Abstract

**Background:**

In clinical and research practice linked to prostate cancer treatment, frequent monitoring of patient health-related quality of life (HRQOL) is essential. Practical and analytic limitations of paper questionnaire data capture may be overcome with the use of self-administered personal digital assistant (PDA) data collection. The objective of this study was to assess the reliability, validity, and feasibility of using PDA in place of paper versions of the International Prostate Symptom Score (IPSS), the Patient Oriented Prostate Cancer Utility Survey (PORPUS), and the International Index of Erectile Function-5 (IIEF-5) in a prostate cancer clinic setting.

**Methods:**

152 participants were randomly assigned to one of three conditions: 1) paper followed by PDA survey; 2) PDA followed by paper survey; or 3) PDA followed by PDA survey. Evaluation included an assessment of data quality (internal consistency, test-retest reliability, response correlation, completeness of data), and feasibility (participation rates, time to completion, preference and difficultly/ease of using PDA).

**Results:**

Internal consistency was similar for both PDA and paper applications. Test-retest reliability was confirmed for PDA repeated administration. Data from paper and PDA questionnaires were strongly correlated. Lower missed item rates were found in PDA administration. 82.8% of participants preferred using the PDA or had no preference. Mean difficulty/ease ratings indicated that participants found the PDA easy to use. Age did not significantly correlate with preference or difficulty.

**Conclusion:**

The results confirm the adaptability of the IPSS, IIEF-5, and the PORPUS to PDA administration. Similarly, the findings of this study support the feasibility of using PDA technology for HRQOL serial data capture in the prostate cancer patient population.

## Background

The long term survival rates and treatment side-effect profiles of most prostate cancer patients combine to emphasize the importance of serial monitoring of health-related quality of life (HRQOL) in advancing clinical care and treatment outcome research. With the advent of prostate specific antigen (PSA) testing, prostate cancer detection rates continue to increase resulting in more and younger men having to cope with the impact of treatment on HRQOL. Specific HRQOL areas of concern for this population of men include sexual, urinary, and bowel dysfunction [1,2]. These side-effects are also known to change over time and can have considerable impact on the patient's emotional, functional, and social well-being [2-4].

Frequent and systematic collection of HRQOL information in the prostate cancer patient population is therefore necessary for informed treatment decision-making, thorough patient care, and comprehensive evaluation of treatment outcome. In collecting this ongoing data, traditional paper questionnaires can be problematic within the clinic setting. For example, if clinical decision-making necessitates input from patient questionnaire data, the paper format can require too much processing time to be helpful under the usual time constraints of the clinical consultation [5]. Furthermore, whether the questionnaire data are electronically scanned or manually entered into a clinic database for analysis, the integrity of the data is vulnerable to data entry errors [6]. These limitations to the traditional paper questionnaire data capture method may be overcome with the use of an electronic data collection system.

Electronic data capture with desktop computers (including touch-screen applications) [7,8], tablets [9], or hand-held personal digital assistants (PDA) [6,10,11] has been shown to improve data quality and allow for immediate and effective data manipulation. Electronic data collection improves data quality by providing software safeguards against entry omission and inconsistent response sets, and by completely eliminating data entry errors at the researcher's level. Moreover, because the data are captured electronically, data manipulation and analysis can be achieved faster than is possible with a paper data capture format. Indeed, results can be immediately scored, displayed and printed, allowing the clinician to review and interpret a patient profile in the company of the patient and discuss possible treatment decisions [6,10,11].

Although there may be a relatively significant start-up cost associated with electronic data collection, costs associated with questionnaires (paper and reproduction), data entry, coding, and cleaning are either eliminated or substantially reduced [6]. Additionally, advances in technology continue to result in reduced costs associated with electronic data collection computer devices and software [10,12].

Handheld PDA devices may be particularly economical and effective for electronic data collection in high traffic clinical healthcare settings [13-16]. PDA technology meets or exceeds the hardware requirements necessary for data collection in most clinical healthcare research. Given the low individual unit cost, multiple PDA units can be deployed at a fraction of the cost of a single desktop, laptop or tablet. The portability of the PDA offers greater flexibility within a clinic setting, less demand for clinic space (e.g. workstation) and, due to PDA energy efficiency, reduced dependence on battery power compared to laptops or tablets.

Moreover, for security purposes, PDA software allows for only a single patient's data to be collected at any one time. The data collected are protected through the use of multiple levels of encryption and a password access system. Once the PDA has been synchronized with the personal computer (PC), the patient self-reported data are purged from the unit. These features ensure that only the current user's data are stored on the PDA and that the data are only accessible to appropriate clinic staff. All other medical data are stored in the Prostate Centre main database and are not directly accessible on either the PC or PDA. The Prostate Centre database is safeguarded through industry standard security and operational protections. Therefore, if a PDA device is lost or stolen, sensitive information will not be accessible.

These advantages notwithstanding, there are some drawbacks to PDA data collection. A potential problem with electronic data collection devices, including the PDA, is that lack of computer literacy may cause some patients to prefer paper forms to computerized versions [17]. The majority of studies, however, document patient acceptance of and preference for using PDAs over paper forms [12,14,17,18]. Patients have also reported difficulty in seeing the questions due to the relatively small display screens in PDA [7,19]. Increasing text font size and restricting the amount of text per screen can overcome this limitation. As well, some patients may be unfamiliar with using PDA devices and may require guidance/training from clinic staff.

Before PDA data collection techniques can be justifiably used for clinical or research purposes in healthcare clinics, this mode of data collection needs to be adequately validated for use with specific measures and patient populations. The aim of this project is to compare self-administered HRQOL measures in a PDA version with a paper version. Evaluation includes an assessment of data quality (internal consistency, test-retest reliability, response correlation, completeness of data), and feasibility (participation rates, time to completion, preference, and difficultly/ease of using PDA). The *unique *relevance of this research is its focus on 1) the responses of *prostate cancer patients *to the PDA data collection system, and 2) the adaptability of the International Prostate Symptom Score (IPSS), the Patient Oriented Prostate Cancer Utility Survey (PORPUS), and the International Index of Erectile Function-5 (IIEF-5) to the PDA format.

## Methods

### Participant selection and study design

Prostate cancer patients at the outpatient clinic of the Prostate Centre, Princess Margaret Hospital, Toronto, between April 1 and July 15, 2006 were considered for participation in the study. Eligible patients were identified by the uro-oncologists and the research team prior to attending each outpatient clinic. Potential participants were approached in the clinic upon registering at reception and the particulars of the study and specific information on the requirements of participation were described. Informed consent was obtained from all eligible patients agreeing to participate. Where possible, reasons for refusing to participate were recorded along with the patient's age and treatment stage (pretreatment or time since treatment). Inclusion criteria included prostate cancer patients (new or follow-up patients) who could read, speak and write English, and who did not have significant hearing or visual impairment. All patients meeting these criteria were invited to participate. This study received approval from the University Health Network Research Ethics Board.

One hundred and fifty-two (n = 152) participants were randomly assigned using a random number generator [20] to one of three HRQOL survey administration conditions: 1) paper survey followed by PDA survey; 2) PDA survey followed by paper survey; or 3) PDA survey followed by PDA survey. Each participant was asked to complete one survey administration at clinic check-in and one after a 30-minute wait period prior to their clinical consultation with the uro-oncologist. The separation in time between completing the two survey administrations allowed for the avoidance of immediate recall responding, and analysis of test-retest reliability of the PDA modality (Condition 3). The alternating paper/PDA survey administrations (Conditions 1, 2) was designed to allow for control of order effects. All participants were given a brief (less than 1 minute) tutorial on using the PDA. Patients were shown how to select a response by tapping the stylus to the PDA, and were instructed on how to enter their date of birth, hospital ID number, and how to proceed to the next screen.

Following completion of two survey administrations (Conditions 1, 2 or 3), participants were given a one-page questionnaire examining the level of difficulty/ease using the PDA and survey format preferences. All participants were asked to rate ease/difficulty by completing a 10-point visual analogue scale (VAS) ranging from "Very Difficult" (0) to "Very Easy" (10). Patients participating in conditions 1 and 2 were then asked to complete a 10-point VAS examining format preferences, ranging from paper questionnaire (0) to PDA questionnaire (10). The cut-off score used to determine ease of use and preference for PDA was 5.1. All participants were given the opportunity to comment on what they liked/disliked about the PDA format.

## Materials

### Health-related quality of life measures

All participants were asked to complete a paper and/or PDA version of the International Prostate Symptom Score (IPSS) [21], the Patient Oriented Prostate Cancer Utility Survey (PORPUS) [22], and the International Index of Erectile Function-5 (IIEF-5) [23]. The IPSS is an 8-item measure of patient urinary voiding function commonly used in prostate cancer research [24-28]. The PORPUS is a prostate cancer specific measure of HRQOL. The 10-item psychometrically validated instrument assesses ten quality of life domains: energy, pain, emotional well-being, support from family/friends, communication with doctor, erectile function, sexual interest, urinary frequency, bladder control and bowel symptoms. The IIEF-5 is a short-form version of the International Index of Erectile Function [29], and is used in clinical settings to assess erectile dysfunction. The questions explore maintenance ability, erection confidence, maintenance frequency, erection firmness and intercourse satisfaction [23]. All of the measures are reliable and valid [21,30,31].

**Figure 1:**
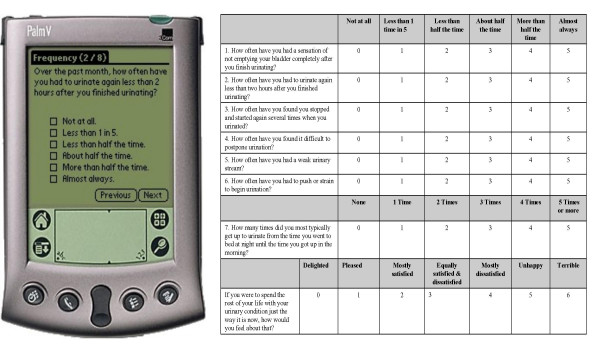
Side by side comparison: PDA and paper/pencil survey.

In order to adapt the IPSS, IIEF-5, and PORPUS to the PDA device, the questions were presented one question per PDA screen. Consequently, in comparison to paper forms, participants completing the PDA version were able to see only one question at a time (Figure 1).

### Personal Digital Assistant PMH Prostate-HRQOL System

**Figure 2:**
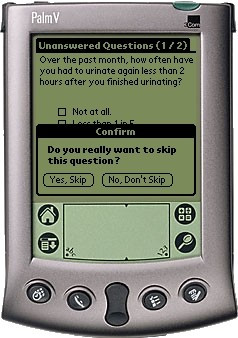
PDA skipped question confirmation screen.

The HRQOL PDA data collection and capture system was developed by the research team. Software was developed for the PDA that allowed a single participant to enter demographics and survey response information. Responses to survey questions were entered by pressing the stylus to the box located beside the chosen statement. Participants had the option of skipping questions by pressing the "Next" button without providing a response. Following completion of the questionnaire, participants who had skipped questions were presented with a screen containing the following statements: "You did not answer on or more of the [survey] questions. Please tap 'Next' and answer the missed questions." The missed questions were then repeated, giving participants the option to complete each of the unanswered questions or to confirm that they have chosen to skip the questions (Figure 2).

Upon completion of the survey, the participant returned the PDA to research staff for data synchronization with a PC. During the synchronization, demographics information was checked against that of known patients stored in a relational database on the PC. If no demographics match was found, the participant was asked to correct his demographics information on the PDA and resynchronize (survey responses were preserved). Otherwise, survey data from the PDA was transferred into the database on the PC, a report was generated for the patient, and the PDA was cleared of data in preparation for the next user.

PDA software was developed in C++ for the Palm Vx series of PDAs. All surveys were administered using Palm Vx PDAs. Synchronization (Palm COM Conduit) and report generation software was written in Visual Basic [32]. Microsoft Access [33] was used as a relational database to transfer data to the Statistical Package for the Social Sciences (SPSS) [34].

### Paper HRQOL Survey

Paper surveys were marked with identification numbers and handed to participants by study investigators. Participants were supplied with a pen and clipboard, and instructed to complete questionnaires in the clinic waiting room. Upon completion of the survey, participants returned the questionnaire package to study investigators. Survey data were entered by an investigator into SPSS [34].

### Statistical methods

Comparisons of characteristics between non-respondents and respondents, and amongst the three experimental conditions were made using chi-square and t-tests. Demographic characteristics of participants are shown in Table 1.

**Table 1:** Comparison of characteristics of respondents by experimental condition

				**Reason for Clinic Visit**		
**Condition**	**N (% of total)**	**Mean Age (S.D.)**	**Age Range (years)**	**Elevated Risk (%)**	**Diagnosis (%)**	**Treatment Follow-up (%)**
1) Paper/PDA	53 (35)	68.7 (10.4)	39 to 89	32 (60)	6 (11)	15 (28)
2) PDA/Paper	53 (35)	67.1 (10.2)	45 to 86	24 (45)	5 (9)	24 (45)
3) PDA/PDA	46 (30)	65.9 (10.3)	47 to 87	23 (50)	3 (7)	20 (43)

### Data quality

Internal consistency of related questionnaire items within administration mode (PDA and Paper) was assessed by Cronbach's alpha. Test-retest reliability within the PDA capture mode and concurrent *validity *examining the reproducibility between the initial administration and repeat administration of the two data capture modes was assessed by the intraclass correlation coefficients (ICC) of a 2-way mixed model reliability model. The ICCs were computed on the total scores for the IPSS, IIEF-5, and for the 10 separate domains of the PORPUS. To investigate possible order effects, an independent sample t-test was used to compare conditions 1 and 2 in terms of variation in scores in the first and second administration. Completeness of data was compared between modes of application on an individual item basis across the three health measures.

### Feasibility

To assess feasibility of using the PDA versus paper administration modes of the HRQOL questionnaires, participation rates, time to completion, and patient preference were recorded in administration conditions 1 and 2, and difficulty with using the PDA administration in administration conditions 1, 2, and 3. Statistical analysis was performed using SPSS [34].

## Results

### Sample characteristics

A total of 216 patients were approached to participate in the study over a 3.5 month period between April 1 and July 15, 2006. Thirty-one patients (22%) declined to participate. The reasons for refusal included: 18 patients (8%) did not want to complete any questionnaires (paper or PDA versions), 5 patients (2%) did not have their eye-glasses, 2 patients (1%) reported time constraints, 1 patient (0.5%) stated that he was uncomfortable with 'electronics', and the remaining 5 patients (2%) did not provide a reason for non-participation. A further 16 patients failed to complete the entire study protocol and were excluded from the analysis. Reasons for partial participation included time constraints and a refusal to complete the questionnaire for a second time. A chi-square analysis revealed these partial respondents were equally represented across experimental conditions. An additional 17 (8%) participants were excluded from the study because clinic wait-times were less than 30 minutes and did not allow for second administration of the questionnaires. Therefore, 152 patients were included in the analysis, 70% of those initially approached about the study. No significant differences were observed between participants and non-participants on age and purpose of clinic visit (elevated risk, cancer diagnosis, treatment follow-up).

As a result of random number assignment, 53 participants completed the paper survey followed by PDA survey (Condition 1), 53 participants completed the PDA survey followed by paper survey (Condition 2), and 46 participants completed the PDA survey followed by another administration of the PDA survey (Condition 3). Between group analysis of participant age and purpose of clinic visit revealed no significant differences (Table 1) amongst the three conditions.

### Data quality

Internal consistency coefficients (Cronbach's alpha) across the IPSS and IIEF-5 revealed excellent internal consistency for both the PDA and paper applications (Table 2).

**Table 2:** Internal consistency coefficients by measure

**Mode of Application**	**IPSS (Cronbach's alpha range)**	**IIEF-5 (Cronbach's alpha range)**
Paper	.78 to .85	.97 to .98
PDA	.85 to .87	.97 to .98

Note: This analysis was not performed on the PORPUS as this scale uses 10 independent questions to measure 10 quality of life domains and is not an appropriate scale for internal consistency analysis.

ICCs used to measure test-retest reliability in Condition 3 for repeated PDA administration of the IPSS, IIEF-5, and the PORPUS quality of life domains are shown in Table 3. The large magnitude correlation coefficients are all highly significant (p < 0.01).

**Table 3:** Intraclass correlation coefficients of PDA administration of questionnaires

**Measure**	**N**	**PDA – 1^st ^Administration Mean (S.D.)**	**PDA – 2^nd ^Administration Mean (S.D.)**	**ICC (C.I.)**
IPSS	46	11.70 (8.96)	9.57 (8.33)	0.960 (0.929–0.978)
IIEF-5	35	14.94 (8.32)	16.06 (8.82)	0.946 (0.896–0.972)
PORPUS				
Pain	44	0.40 (0.65)	0.42 (0.78)	0.714 (0.532–0.833)
Energy	46	1.18 (0.97)	1.11 (0.97)	0.976 (0.958–0.987)
Social support	46	0.30 (0.72)	0.11 (0.38)	0.928 (0.874–0.960)
Communication with doctor	45	0.17 (0.42)	0.22 (0.42)	0.589 (0.360–0.751)
Emotional well-being	46	0.80 (0.94)	0.59 (0.91)	0.910 (0.844–0.949)
Urinary frequency/urgency	44	1.19 (0.91)	1.15 (0.94)	0.867 (0.768–0.925)
Leaking urine	45	1.00 (1.19)	0.98 (1.27)	0.840 (0.726–0.909)
Sexual function	41	1.72 (1.54)	1.60 (1.62)	0.973 (0.950–0.986)
Sexual interest	42	1.25 (1.47)	1.05 (1.38)	0.878 (0.785–0.933)
Bowel problems	43	0.21 (0.45)	0.20 (0.46)	0.873 (0.777–0.929)

*All correlations are significant at p ≤ 0.01

ICCs reveal that the total scores by patients completing paper and PDA questionnaires were strongly correlated (Table 4a and 4b). This provides evidence of concurrent validity across administration modalities. The correlation coefficients reported are all highly significant (P < 0.01).

**Table 4a:** Concurrent validity across administration modalities – Condition 1 (Paper/PDA)

**Measure**	**Paper: Mean (S.D.)**	**PDA: Mean (S.D.)**	**N1**	**ICC (C.I.)**
IPSS	10.9 (6.88)	9.8 (6.62)	52	0.901 (0.834–0.942)
IIEF-5	11.39 (8.25)	12.39 (7.73)	41	0.963 (0.931–0.980)
PORPUS				
Pain	0.41 (0.77)	0.43 (0.75)	53	0.92 (0.865–0.953)
Energy	1.5 (0.91)	1.53 (0.89)	53	0.898 (0.830–0.940)
Social support	0.25 (0.69)	0.29 (0.72)	52	1 (1.00–1.00)
Communication with doctor	0.26 (0.52)	0.42 (0.949)	52	0.38 (0.122–0.590)
Emotional well-being	0.73 (0.86)	0.74 (0.74)	53	0.78 (0.647–0.867)
Urinary frequency/urgency	1.1 (0.75)	1.08 (0.76)	52	0.818 (0.702–0.891)
Leaking urine	0.88 (0.8)	1.23 (1.07)	53	0.551 (0.332–0.714)
Sexual function	2.17 (1.49)	2.06 (1.42)	51	0.924 (0.871–0.956)
Sexual interest	1.53 (1.42)	1.67 (1.38)	50	0.934 (0.886–0.961)
Bowel problems	0.4 (0.59)	0.34 (0.52)	53	0.933 (0.886–0.961)

*All correlations are significant at p ≤ 0.01

**Table 4b:** Concurrent validity across administration modalities – Condition 2 (PDA/Paper)

**Measure**	**Paper: Mean (S.D.)**	**PDA: Mean (S.D.)**	**N1**	**ICC (C.I.)**
IPSS	10.88 (7.5)	9.08 (6.41)	53	0.82 (0.707–0.892)
IIEF-5	11.18 (7.5)	12.27 (8.48)	39	0.86 (0.751–0.923)
PORPUS				
Pain	0.47 (0.72)	0.46 (0.75)	52	0.696 (0.524–0.814)
Energy	1.46 (0.95)	1.36 (0.96)	53	0.88 (0.800–0.929)
Social support	0.33 (0.86)	0.32 (0.85)	53	0.987 (0.977–0.992)
Communication with doctor	0.47 (0.70)	0.36 (0.65)	53	0.922 (0.868–0.954)
Emotional well-being	0.86 (0.97)	0.90 (0.93)	52	0.958 (0.928–0.976)
Urinary frequency/urgency	1.24 (0.97)	0.92 (0.9)	52	0.723 (0.562–0.831)
Leaking urine	1.1 (1.27)	0.87 (1.1)	52	0.869 (0.782–0.923)
Sexual function	2.19 (1.59)	2.17 (1.57)	46	0.992 (0.985–0.995)
Sexual interest	2.02 (1.53)	1.85 (1.63)	47	0.93 (0.878–0.961)
Bowel problems	0.52 (0.78)	0.39 (0.64)	50	0.878 (0.794–0.929)

*All correlations are significant at p ≤ 0.01

An independent sample t-test was used to compare conditions 1 and 2 in terms of the variation in scores inputted in the first and second administration. Non-significant t-scores suggest that there was no order effect. A final aspect of data quality that was measured included a comparison of completeness of data collected via the two modes of administration. Examination of individual item completion in Condition 1 revealed that when completing the IPSS, IIEF-5 and PORPUS in the paper format, 10%, 27.7%, and 7.5% of respondents failed to complete at least one question. In comparison, when completing the IPSS, IIEF-5, and PORPUS in the PDA format, 0%, 13.6%, and 7.5% of respondents failed to complete at least one question. Similarly, the same analysis in Condition 2 revealed that 11.3%, 17.0%, and 5.1% of the items were missed in the paper administration version of the IPSS, IIEF-5, and PORPUS, respectively. In contrast only 0%, 6.8%, and 5.7% of the items were missed on the PDA administration versions of the IPSS, IIEF-5, and PORPUS, respectively. Finally, the analysis of Condition 3 revealed exactly the same results for the first and second administration of the questionnaire using the PDA. Missed item rates for the IPSS, IIEF-5, and PORPUS were 0%, 0%, and 8.7% respectively.

### Feasibility

A Pearson chi-square was used to assess possible differences in level of participation (full, partial, decline) across experimental condition (1, 2, 3) for all 216 patients approached for participation in this study. No significant differences were found. As well, in comparing mode of application, participants were able to complete the survey significantly faster using the PDA. Mean times for completion using the paper survey for conditions 1 and 2 were 12.5 minutes, and 10.2 minutes, respectively, compared to 11.2 minutes and 8.6 minutes respectively using the PDA format.

Preference for using paper or PDA modes of application was measured using a 10-point VAS, ranging from paper preference to PDA preference. Results of the analysis revealed that 58.6% of patients preferred using the PDA format, 24.2% had no preference, and 17.2% preferred the paper format. To determine if age had an impact on preference, a Pearson correlation coefficient was computed between age and the mode preference scale. The correlation coefficient was weak (r = 0.16, p = 0.12) and not statistically significant. In a similar fashion, difficulty of use of the PDA administration was also measured using a 10-point VAS. The mean score was 8.6. Again, there was a weak and non-significant correlation between age and scores on the visual analogue difficulty scale (r = -0.15, p = 0.08).

All participants were provided with a brief tutorial on using the PDA. Following the tutorial, a research assistant documented the frequency and content of questions asked by participants while using the PDA. The majority of patients (76%) were able to complete PDA administered questionnaires without requiring further instruction. Twenty-nine (19%) patients were able to complete the PDA administered questionnaire by presenting with 1 or 2 follow-up questions or concerns, and the remaining 7 (5%) patients presented 3 or more inquiries. Questions or concerns included difficulty recording answers on the PDA as a result of stylus-device insensitivity. Other inquiries included how to skip questions, and how to correct errors entering hospital ID or date of birth. Four patients required assistance with the paper questionnaire.

## Discussion

### Data quality

The results of this study showed that PDA administration of the IPSS, IIEF-5, and the PORPUS was psychometrically comparable to paper administration. Internal consistency analysis revealed identical coefficients (Cronbach's alphas) for the IIEF-5 under both modes of application, and slightly better coefficients for PDA administration of the IPSS compared to paper survey. The test-retest reliability coefficients should be high given that we do not expect the participant's 'state' to change over the 30-minute testing interval. The generally accepted test-retest reliability coefficient under these circumstances is above 0.70 [35]. Our analysis found coefficients for the IPSS, IIEF-5, and nine out of ten domains of the PORPUS exceeded the 0.70 cut-off. "Communication with Doctor" was the one domain of the PORPUS that produced only a moderate coefficient of 0.59. This moderate value is similar to the test-retest coefficient reported for the paper version of the PORPUS [30] and is therefore not a direct result of the PDA format. However, in this study, given that the participants did not have any direct contact with their physicians in the interval between testing, this moderate correlation is an unexpected result. The mean score for the "Communication with Doctor" question increased from the first to second administration, from 1.7 (S.D. = 0.42) to 2.2 (S.D. = 0.41) respectively, and represents a self-reported decrease in communication with doctor. It is possible that this reported decline in communication is explained by participant frustration associated with waiting a minimum of 30 minutes to see the physician.

In conditions 1 and 2, we made direct comparisons of data collected by the paper and PDA formats. Our ICCs should be high across administration modes given that our analysis of test-retest reliability suggests scores are stable over the testing period. Once again, in conditions 1 and 2 we found strong and significant ICCs between modalities for the IPSS, IIEF-5, and the PORPUS. Exceptions were found in our analysis of Condition 1 in which the PORPUS domains, "Communication with Doctor" and "Leaking Urine" revealed moderate ICCs of 0.38 and 0.55 respectively. It is not clear why these ICCs are only moderate and different than those found in our analysis of Condition 2 (0.92 and 0.87 respectively). Our analysis of order effects, however, revealed that the difference in mean scores for the "Communication with Doctor" and the "Leaking Urine" questions were non-significant (p = 0.44 and p = 0.13, respectively). Unfortunately, closer examination of participant responses in both conditions did not lead to a further understanding of this domain-specific deviation. The overall strong ICCs, however, suggest that researchers and clinicians can be confident that similar data can be collected using both modes. Our findings are similar to findings in previous literature where there continues to be very little evidence of mode effects in data capture [36-38].

Our findings also support previous literature reporting that PDA administration can enhance completeness of data collection. No missed items were found in the PDA mode collection of IPSS data, while 10 to 11% of the paper surveys contained missed items. The number of missed items for the PORPUS was low and virtually identical under both conditions of data collection. For the IIEF-5 the number of missed items in conditions 1 and 2 was high, although the PDA mode missed-item rate was less than half that found using paper questionnaires. The overall elevated number of missed items on the IIEF-5 is likely due to the sensitive topic of the questionnaire. Our experience in the serial collection of over 3500 paper IIEF-5 questionnaires in the Prostate Centre since 2005 (missed-item rate of 17.6%) suggests that the overall missed-item rate in this study is not unusual. It appears from the response pattern, patients with little or no erectile functioning complete questions 1 and/or 2 and leave the remaining 3 questions unanswered. The reason for our finding of the advantage of the PDA mode in collecting sensitive data is not clear, although this benefit has been reported in previous literature comparing self-administered electronic to paper sensitive data capture [39,40]. It is possible that the single-question per screen in the PDA administration along with skipped question reminders (Figure 2) encourages patients to respond to each question in order to progress to the next screen. As well, the more "private" or "concealed" nature of the PDA (i.e. it is difficult for others sitting adjacent to the participant to see the PDA screen) may support participants in feeling more comfortable spending time to complete the entire questionnaire. Overall, this increased data collection has a direct beneficial impact on the quality of the data collected.

### Feasibility

Our findings strongly support the utility and acceptance of PDA data capture in a clinically diverse population of prostate cancer patients. In examining patient preference for mode of application, 54.6% of patients preferred PDA questionnaire administration and 24.2% had no preference. Thus, 82.8% of the prostate cancer patients attending our clinic were willing participants to the adaptation of using PDA technology. Positive comments regarding PDA administration included: "the PDA is fast and efficient" and "using the PDA will help save paper." Some study participants (17.2%), stated that they preferred completing paper questionnaires. The main reason these patients provided for preferring paper surveys was that they were "used to them." Another common reason was that these patients found the stylus awkward to use. Interestingly, this was not because the patients found the stylus manually difficult to manipulate, but because they reported being frustrated by encountering difficulties in recording answers on the PDA as a result of stylus-device insensitivity. It appears that these reasons for non-preference can be accommodated through brief training and adjustment in existing technology. In this regard, we can also be optimistic about successful use of PDA data collection within this population given that the majority of participants in this study rated the PDA device as very easy to use. Given the clinical and research benefit of PDA data capture and that the device is readily acceptable to prostate cancer patients, there appear to be few reasons not to take advantage of this technology in prostate cancer clinics requiring continuous health status monitoring.

## Design Issues

### Selection bias

It is important to acknowledge the potential impact of selection bias in this type of research. Selection bias can occur in two ways: 1) self-selection of individuals to participate in an experimental study; and 2) selection of samples that may introduce systematic bias to the generalizability of the findings. Both of these selection biases represent a challenge to the validity of this study. First, a specific concern in studying the prostate cancer population was that older patients may be less familiar or comfortable with computer technology, and therefore these patients may decline to participate.

To investigate this potential source of bias, we asked about the age of patients who declined to participate. Statistical comparison of the mean age of non-participants to participants was non-significant. As well, the age of participants in the trial indicates representation across a broad age-range. We were also concerned that patient distress, which may vary according to a patient's reason for his clinic visit (e.g. elevated risk, diagnosis and treatment decision-making, or treatment follow-up), could influence willingness to participate in research. Once again, comparisons of non-participants and participants regarding their reasons for attending the Prostate Centre revealed no significant differences. Second, recruitment only included patients treated by an urologist-co-investigator in a prostate clinic of a large urban hospital. This sampling method limits the degree of generalization of our results and may not reflect outcomes in other settings.

In summary, we acknowledge the common difficulty of selection bias in this type of research and we attempted to control for obvious individual patient selection bias, while recognizing the limits of generalization imposed by the sample. Comparisons between participants and non-participants however, suggest that the 152 participants in this study are representative of patients typically seen in our specialty prostate cancer treatment centre. Additionally, analysis of level of participation (full, partial, and decline) did not find a significant difference between conditions, suggesting that we can be confident that selection bias by condition was controlled for by random group assignment.

### Test-Retest Reliability

Test-retest reliability of the PDA version of the questionnaires was evaluated in Condition 3. In determining the time interval between questionnaire administrations, two factors were considered; 1) the longest time interval possible within the framework of the clinic; and 2) the potential confounding nature of participant-physician contact. The majority of patients attending the clinic wait at least 30 minutes prior to seeing the physician; thus, we attempted the second questionnaire administration 30 minutes after the first administration but prior to any participant-physician contact. We would have preferred a longer interval between testing, however we feel the 30-minute separation achieves the best possible balance between the risks of immediate recall versus the potential influence of extraneous factors during the test-retest time interval.

## Conclusion

The major goals of this study were two-fold: 1) to assess the reliability and validity of PDA administration of the International Prostate Symptom Score (IPSS) [21], the International Index of Erectile Function-5 (IIEF-5) [41], and the Patient Oriented Prostate Cancer Utility Survey (PORPUS) [22]; and 2) to determine the feasibility of serial data capture through patient completed PDA administered HRQOL questionnaires.

The results of this study confirm the adaptability of the IPSS, IIEF-5, and the PORPUS to PDA administration. Analyses of data quality, including internal consistency, test-retest reliability, and concurrent paper-PDA mode suggest that the psychometric properties of these questionnaires are maintained under PDA application. Furthermore, data quality of the PDA mode was superior to the paper mode due to increased levels of data completion. The findings of this study also support the feasibility of using PDA technology for data capture in the prostate cancer patient population. Assessment of study participation rates, time to completion, patient preferences, and difficulty of use revealed that PDA administration met or exceeded the practicability of regular paper administration.

Patient acceptability combined with valid and reliable data capture of the PDA device suggest that PDA technology may be efficiently and effectively used by both researchers and clinicians in the study and treatment of prostate cancer.

### Future directions

A future research direction is the examination of the clinical impact of PDA produced real-time patient-physician feedback. A significant advantage to the use of PDA administered surveys is the capability to produce an immediate print-out of data provided in the survey. Research reveals that measurement of patient HRQOL can be used in clinical practice to monitor physical and psychological functioning, inform treatment decision-making, and improve care [42]. PDA administration makes real-time patient-physician feedback practicable even in high-traffic clinics. One recent study found that oncology patients receiving immediate feedback of HRQOL information report their physicians enquire about daily activity and emotional problems more often than without computerized results. Furthermore, physicians indicate that HRQOL information improves communication with patients and can assist in treatment or disease management decisions [42]. Another study indicated cancer patients experience clinically meaningful improvements in HRQOL after three sessions of using immediate feedback print-outs [43]. Perhaps more importantly, real-time review of HRQOL information helps physicians identify patients experiencing significant reductions in quality of life, thus promoting rapid assessment of patient health and promoting intervention when necessary [44].

## Competing interests

The author(s) declare that they have no competing interests.

## Authors' contributions

Authors' contributions to this study include: conception and design (AGM, KLC, PR, LJ, RN & JT); analysis and interpretation of data (AGM, KLC, JI & DSM); drafting of the manuscript (AGM, KLC & JI); and revising the manuscript for intellectual content (AGM, JI, PR, LJ, RN & JT). All authors read and approved the final manuscript.
